# Exploring the Use of Artificial Intelligence in the Management of Prostate Cancer

**DOI:** 10.1007/s11934-023-01149-6

**Published:** 2023-02-18

**Authors:** Timothy N. Chu, Elyssa Y. Wong, Runzhuo Ma, Cherine H. Yang, Istabraq S. Dalieh, Andrew J. Hung

**Affiliations:** Center for Robotic Simulation & Education, Department of Urology, USC Institute of Urology, University of Southern California, Catherine & Joseph Aresty1441 Eastlake Avenue Suite 7416, Los Angeles, CA 90089 USA

**Keywords:** Artificial intelligence, Machine learning, Prostate cancer, Radiomics, Pathomics

## Abstract

**Purpose of Review:**

This review aims to explore the current state of research on the use of artificial intelligence (AI) in the management of prostate cancer. We examine the various applications of AI in prostate cancer, including image analysis, prediction of treatment outcomes, and patient stratification. Additionally, the review will evaluate the current limitations and challenges faced in the implementation of AI in prostate cancer management.

**Recent Findings:**

Recent literature has focused particularly on the use of AI in radiomics, pathomics, the evaluation of surgical skills, and patient outcomes.

**Summary:**

AI has the potential to revolutionize the future of prostate cancer management by improving diagnostic accuracy, treatment planning, and patient outcomes. Studies have shown improved accuracy and efficiency of AI models in the detection and treatment of prostate cancer, but further research is needed to understand its full potential as well as limitations.

## Introduction

Artificial intelligence (AI) and machine learning (ML) are rapidly advancing fields that have the potential to revolutionize many industries, including medicine. AI involves the development of intelligent systems to perform tasks that typically require human intelligence, such as recognizing patterns and making decisions. AI performance is driven by ML, which harnesses algorithms and statistical models to automatically improve system performance on a specific task through experience. Over the last decade, AI has started to become increasingly integrated into medicine. Specifically, in the field of urology, AI is being tested and implemented as a tool to aid in the diagnosis and treatment of prostate cancer. AI-driven techniques are highly appealing because they can quickly analyze large amounts of data, such as medical images and tissue samples, to identify patterns and make predictions about the likelihood of cancer [1]. In addition, AI-based techniques show the potential to increase the accuracy of prostate cancer diagnosis and improve treatment plans for patients. In this review, we explore recent AI advances in the diagnosis, prognosis, and treatment of localized prostate cancer. We share key studies and the impact they bring to each of these areas and highlight potential avenues for future research.

## Methods

A comprehensive review of the current literature was performed using the PubMed-Medline database up to 2023 using the term “urology” combined with the following terms: “prostate cancer” and “artificial intelligence.” To capture recent trends in ML and DL applications, our search was focused on articles published within the last 4 years and originally published in English. Review articles and editorials were excluded. Publications relevant to the subject and their cited references were retrieved and appraised independently by 2 authors for inclusion in the final manuscript.

## Diagnostics

### Radiomics

Analysis of cross-sectional radiographic images, like those produced by CT scans or MRI, is used to identify complex patterns, a task that AI can be trained to perform quickly, accurately, and consistently. The extraction of quantitative features from medical imaging, otherwise known as radiomics, has been studied for use in clinical settings [2]. Specifically, in the context of prostate cancer, ML algorithms can automatically extract qualitative information regarding tumor features such as size, shape, texture, and intensity from medical imaging data. This quantitative data can be delivered back to a urologist to provide objective, data-driven insights into the characteristics and behavior of prostate tumors and offer insight into monitoring response to treatment and prediction of outcomes. This allows clinicians to adjust treatment plans as needed with less delay (Table 1).

**Table 1:** Radiomics

**Reference number**	**Reference**	**Year**	**Population**	**Modality**	**Outcome/prediction**	**AUC/performance**
[3]	Antonelli et al.	2019	164 male patients (119 PZ, 45 TZ) training, 30 test set	mpMRI	Gleason 4 component	Peripheral zone model: AUC 0.83 Transitional zone model: AUC 0.75
[4]	Fehr et al.	2015	147 male patients	mpMRI	Gleason score	Distinguished GS 6 (3 + 3) and GS ≥ 7 cancers with 93% accuracy Distinguished GS 7 (3 + 4) from GS 7 (4 + 3) with 92% accuracy
[6•]	Schelb et al.	2019	250 male patients training set, 62 male patients test set	mpMRI	csPCa	Sensitivity 88% and specificity 50% for PI-RADS ≥ 4 vs U-Net
[7]	Hectors et al.	2021	188 male patients training set, 52 male patients test set	mpMRI	csPCa	AUC 0.76 (*p* = 0.022) for prediction of csPCa in test set using T2WI radiomics features
[8]	Min et al.	2019	187 male patients training set, 93 male patients test set	mpMRI	csPCa vs ciPCa	Training set: AUC 0.872 Test set: AUC 0.823
[9]	Woźnicki et al.	2020	191 male patients	mpMRI	csPCa vs ciPCa	Differentiation of malignant from benign prostatic lesions: AUC 0.889 csPCa from ciPCa: AUC 0.844
[10]	Winkel et al.	2020	48 male patients	bpMRI	PCa screening	Sensitivity 87%, specificity 50%, *k* = 0.42 PI-RADS 3: 43% detection PI-RADS 4: 73% detection PI-RADS 5: 100% detection
[11]	Varghese et al.	2019	68 male patients	mpMRI	To identify the best performing classifier for PCa risk stratification	AUC 0.92
[12]	Cysouw et al.	2021	76 male patients	PSMA PET-CT	Metastatic disease or high-risk features (LNI, distant metastasis, and Gleason score)	LNI AUC 0.86, nodal or distant metastasis AUC 0.86, Gleason score AUC 0.81, and ECE 0.76
[13]	Papp et al.	2021	52 male patients	PET/MRI	Prostate lesion-specific low-vs-high risk	Low-vs-high risk lesion prediction model AUC: 0.86 Biochemical recurrence model AUC: 0.9 Overall patient risk model AUC: 0.94
[14]	Akatsuka et al.	2022	772 male patients, 2899 ultrasound images	Ultrasound	High-grade PCa	AUC using clinical data 0.691 AUC using clinical data and ultrasound imaging data 0.835
[15]	Wildeboer et al.	2020	48 male patients	Ultrasound	Multiparametric classification of PCa	The multiparametric classifier reached a ROC-AUC of 0.75 and 0.90 for PCa and Gleason > 3 + 4 significant PCa, respectively
[16]	Khosravi et al.	2021	400 male patients	MRI	Benign vs cancerous, high-risk vs low-risk PCa	Cancer vs benign: 0.89 (95% confidence interval [CI]: [0.86–0.92]) High- vs low-risk: 0.78 (95% CI: [0.74–0.82])

*bpMRI* biparametric MRI, *mpMRI* multi-parametric MRI, *PSMA* quantitative prostate-specific membrane antigen, *ECE* extracapsular extension, *LNI* lymph node invasion, *SWE* shear-wave elastography, *DCE-US* dynamic contrast-enhanced ultrasound, *ROC-AUC* region-wise area under the receiver operating characteristics curve

#### Radiomics and Gleason Score

The Gleason grading system is based on the microscopic appearance of cancer cells and remains the gold standard for grading prostate cancer. Traditionally, tissue samples are obtained from the prostate gland during a biopsy or after surgery which can have associated complications (such as bleeding, infection, and urosepsis). Multiple studies have explored whether radiomic features extracted from MRI scans can be used to predict the Gleason score as an alternative to traditional methods. Antonelli et al. performed a study in which machine learning classifiers were trained on various MRI features to classify and compare transitional and peripheral zone prostate tumors. The sensitivity of their peripheral zone model was 0.93, compared to an average sensitivity of 0.72 for three radiologists [3]. In another study, Fehr et al. used a support vector machine classifier trained on apparent diffusion coefficients and T2-weighted MRI-based texture features from a cohort of 217 men to accurately distinguish between Gleason scores 6 and 7 or higher, with an area under the curve (AUC) of 0.93 in both the peripheral and transition zones [4]. The ability to use MRI images to predict prostate tumor Gleason scores may provide information that is useful in guiding treatment while also reducing the risk of complications associated with more invasive means of tissue sampling.

#### Clinically Significant Prostate Cancer vs Clinically Insignificant Prostate Cancer

Multiparametric prostate MRI (mpMRI) has become more widely used in the diagnosis of clinically significant prostate cancer. The Prostate Imaging Reporting and Data system (PI-RADS) is an international standard for acquiring, interpreting, and reporting MRI images of the prostate [5]. PI-RADS 1 and 2 lesions usually indicate lower chances of clinically significant cancer, while PI-RADS 4 and 5 lesions usually indicate higher chances of clinically significant prostate cancer. Category 3 lesions pose challenges to clinicians and radiologists alike due to the ambiguity of this designation in terms of clinically significant or insignificant prostate cancer. There is growing evidence that ML models can rival the performance of clinical radiologists in the assessment of PI-RADS lesions [6•]. These models may offer the means to clearly distinguish between clinically significant prostate cancer (csPCa) and clinically insignificant prostate cancer (ciPCa). Hectors et al. used machine learning to construct and cross-validate a model using radiomic features from T2-weighted imaging of PI-RADS 3 lesions to identify clinically significant prostate cancer [7]. Using a training set of 188 subjects and a test set of 52 subjects, they were able to train a random forest classifier with an AUC of 0.76 for predicting csPCa in the test set. In another study, Min et al. used nine radiomic features to train a LASSO algorithm that accurately distinguished between csPCa and ciPCa, with an AUC of 0.82, sensitivity of 0.883, and specificity of 0.753 in the training set, and an AUC of 0.823, sensitivity of 0.841, and specificity of 0.727 in the test cohort [8]. Woznicki et al. developed predictive machine learning models and compared them to PI-RADS-based assessments by radiologists to determine their ability to identify malignant versus benign prostate cancer and csPCa versus ciPCa [9]. In their test cohort, they achieved an AUC of 0.889 with their ensemble model in differentiating between malignant and benign prostate lesions. Their model was also able to achieve an AUC of 0.844 in distinguishing csPCa and ciPCa.

#### Risk Stratification

Risk stratification is used to assess the likelihood of cancer progression and helps determine the appropriate course of treatment based on this risk. Using existing tools, patients can be classified as having low-, intermediate-, or high-risk prostate cancer with different clinical implications for each. Appropriate treatment depending on risk level may include active surveillance, surgery, radiation therapy, and/or hormone therapy, given the differences in the importance of an accurate risk stratification method. One application of ML in risk stratification was demonstrated by Winkel et al., who investigated whether machine learning algorithms in combination with biparametric imaging could accurately detect and classify prostate lesions in asymptomatic men [10]. They trained a model using a cohort of 48 men, 38 of whom had high-risk lesions while 10 were lesion-free. The model was able to identify and classify 100% of the highest-risk lesions (PI-RADS category 5) and 73% of the intermediate-risk lesions (PI-RADS category 4). Varghese et al. developed a quadratic kernel-based support vector machine (SVM) algorithm that used 110 radiomic features to distinguish between high-risk and low-risk prostate cancer [11]. The algorithm achieved a positive predictive value of 0.57 and a negative predictive value of 0.84 when tested on a study cohort of 68 patients who were divided into high-risk and low-risk groups. Cysouw et al. used pre-operative PET-CT scans from 76 patients with intermediate- to high-risk PCa, to train random forest models to predict lymph node metastasis, Gleason score > 8, and extracapsular extension [12]. This ML model was capable of predicting lymph node invasion (AUC 0.86 ± 0.15, *p* < 0.01), lymph node/distant metastasis (AUC 0.86 ± 0.14, *p* < 0.01), Gleason score > 8 (AUC 0.81 ± 0.16, *p* < 0.01), and extracapsular extension (AUC 0.76 ± 0.12, *p* < 0.01) with high accuracy [12]. Papp et al. used a combination of PET-MRI to predict low versus high-risk lesions, biochemical recurrence, and overall patient risk [13].

#### Ultrasound

Ultrasound is a cost-effective and efficient tool that offers insightful information in the context of prostate cancer. There have been recent attempts to apply ML analysis to ultrasound imaging data in high-grade prostate cancer [14, 15]. The model demonstrated by Akatsuka et al. used a combination of ultrasound images and clinical data to achieve an AUC of 0.835 in the detection of high-grade cancer (Gleason grade group ≥ 4) [14]. Wildeboer et al. leveraged ultrasound imaging and a random forest-based classifier to improve the localization of Gleason > 3 + 4 prostate [15]. The application of US imaging is still an emerging area of research within radiomics that has the potential to supplement more established imaging modalities such as MRI and CT.

#### Mixed Modality

Multimodal ML-based approaches blend the strengths of different imaging modalities to optimize the visualization of anatomic structures (i.e., CT or MRI) with other modalities that emphasize function (i.e., PET, US). Khosravi et al. utilized an AI-driven approach that combined MRI and histopathologic data from biopsy reports to increase the accuracy of PI-RADS scoring [16]. Another study utilized mpMRI as a prescreening test before TRUS-biopsy among men with clinical suspicion of prostate cancer.

### Pathomics

Pathomics involves using AI to analyze tissue samples, such as biopsy samples, to identify prostate cancer at the molecular level [17]. The Gleason grading scale remains the strongest predictor of prostate cancer prognosis. ML systems provide an opportunity to reduce inter-observer variability, improve diagnostic accuracy, and streamline the process of grading prostate biopsies. Automated Gleason grading has the potential to produce more objective and reproducible score assignments [18], while performing at a level similar to pathologists, proving a reliable tool for screening or an additional layer of verification [19•]. Kott et al. tested an AI-based system for detecting prostate cancer which yielded 91.5% accuracy in classifying slides as either benign or malignant, and 85.4% accuracy in finer classifications of benign vs Gleason 3 vs 4 vs 5. The model experienced the greatest difficulty in differentiating between Gleason 3 and 4, and Gleason 4 and 5 [20]. Gleason 4 pathology posed a challenge for automated detection methods when it presented as small or fused glands without lumina. Automatic detection of Gleason pattern and grade groups classification using a convolutional neural network (CNN) resulted in a 90% accuracy in differentiating between Gleason scores 3 and 4 [21]. Another algorithm based on CNN and ML showed accuracy in detecting Gleason 3 and 4, but to a lesser degree for Gleason 5 [22]. AI computing power has also been harnessed to transform two-dimensional histopathology slides into 3D computational models in an effort to improve risk stratification for patients with prostate cancer [23]. In a study conducted by da Silva et al., the implementation of AI-based systems in histopathology was shown to reduce analysis and diagnostic time by approximately 65.5% and aid in the identification of prostate cancer in patients who were not previously diagnosed by 3 histopathologists [24]. A population-based diagnostic study trained an AI system to reliably detect and grade prostate cancer in needle core biopsies comparable to expert pathologists. AI systems with clinically acceptable accuracy could alleviate the demand on pathologists by screening out benign biopsies and automating the measurement of cancer length in malignant biopsies [25]. One important caveat of AI-based techniques is the potential for bias in classification performance due to patch-wise comparison and training on a single expert data. Nir et al. found that this can be ameliorated by using patient-based cross-validation and training on multiple expert data [26]. Efforts to understand and reduce bias are valuable to improving AI algorithms.

## Treatment

### Surgical Skill Assessment

Conventionally, surgical skill evaluation is performed manually by human graders, which is time-consuming and prone to observer biases [27]. AI provides an ideal solution to both issues. Utilizing enriching data (i.e., surgical videos and instrument kinematics) derived from surgery, AI is starting to show promise in surgical assessment [28]. AI can be combined with surgical metrics to assess surgeons. Hung et al. used kinematic metrics derived from surgical robots (e.g., path length and velocity of instruments) to distinguish surgeons’ skill levels and predict surgical outcomes after robotic assisted radical prostatectomy (RARP) [29]. Empowered by AI, such models were able to predict surgeons’ experience level, short-term outcomes such as length of hospital stay, and long-term outcomes such as continence recovery [29–32]. Interestingly, AI models using surgical performance represented by automated performance metrics (APMs) better predict surgical outcomes than only using a surgeon’s prior experience. This brings into question the view of a surgeon’s experience as the presumed gold standard for performance proxy [30]. AI-aided vision recognition has also been used to assess surgical performance directly from surgical videos. Khalid et al. developed a machine learning model using the JIGSAWS video footage to accurately detect surgical actions (needle passing, suturing, knot tying) and predict performance levels (novice, intermediate, expert) [33]. Baghdadi et al. described machine learning analysis of color and texture to recognize anatomical structures during pelvic lymph node dissection and predict dissection quality. The automated skill assessment output from their model compares favorably with manually scored expert ratings of lymph node dissection quality (83.3% accuracy), setting the stage for further evaluation of these training tools [34]. Hung et al. trained a deep learning model to give robotic suturing assessments in four domains—needle positioning, needle entry angle, needle driving, and needle withdrawing [35•].

Another innovation in recent years is that AI models can automatically recognize basic phases in surgery such as different surgical steps. This can significantly reduce the time associated with surgical video review and help maintain a good surgical library for educational purposes. For example, Zia et al. applied a machine learning model to automate the segmentation of RARP into 12 surgical steps. Compared with expert annotations, the model correctly annotated most RARP steps with less than 200 s of error [36].

AI has even demonstrated the ability to recognize single instrument movements in a surgical procedure and classify them into different categories, namely, surgical gestures. Luongo et al. trained deep-learning-based computer vision algorithms to identify different suturing gestures during vesicourethral anastomosis of RARP with an AUC of 0.87 [37]. Kiyasseh et al. trained a multi-purpose model which could not only recognize surgical gestures (both dissection and suturing), but could also evaluate surgical quality for multiple different steps of RARP [38]. Furthermore, by breaking down surgery into individual surgical gestures, differences in gesture usage have been found between experienced surgeons and trainees, providing new insights for both surgical assessment and training. By giving surgical gesture sequences to AI models, a study has been able to predict patients’ erectile function recovery in the long term [39•]. This opens up a new avenue for surgical assessment and training (Table 2).

**Table 2:** Skill assessment

**Reference number**	**Reference**	**Application**	**Population**	**Performance**
[29]	Hung et al.	Automated assessment of surgical performance and predicting clinical outcomes after RARP	78 RARP cases	Using the kinematic data from the surgical robot to represent surgeon performance, random forest-50 had 87.2% accuracy in predicting LOS (≤ 2 days vs > 2 days)
[30]	Hung et al.	Automated prediction of urinary continence recovery after RARP using kinematic data, followed by assessment of surgeon’s historical patient outcomes	100 contemporary + 493 historical RARPs cases	DL model obtained a C-index of 0.6 and an MAE of 85.9. Surgeon group with better kinematic data performance had superior continence rates at 3 and 6 months postoperatively (47.5 vs 36.7%, *p* = 0.034; 68.3 vs 59.2%, *p* = 0.047)
[31]	Trinh et al.	Automated prediction of urinary continence recovery after RARP evaluated with patient and treatment factors, kinematic data, and technical skills of surgeon	115 RARP cases	Best models were produced by technical skills (C index: CoxPH − 0.695, DeepSurv: 0.708). Model performance for posterior/anterior VUA outperformed other steps (C index 0.543–0.592). Needle driving achieved top-performing models (C index 0.614–0.655)
[32]	Lee et al.	Using ML to predict positive surgical margins with surgeon performance (represented by kinematic data) and clinical factors	236 RARP cases	Model achieved AUC of 0.74. When assessing only clinical factors or kinematics, the model obtained AUC 0.72 and 0.64, respectively
[33]	Khalid et al.	Using ML models to assess surgical characteristics and performance	103 video clips of surgical procedures performed by 8 surgeons from JIGSAW dataset (benchtop models)	For surgical actions and surgical skill level of operators, deep machine learning yielded a mean precision of 0.97 and 0.77, and a mean recall of 0.98 and 0.78, respectively
[34]	Baghdadi et al.	Automated assessment of surgical performance in PLND	20 PLND video recordings	Model achieved an accuracy of 83.3% in predicting the expert-based PLACE scores
[36]	Zia et al.	Automating recognition of individual surgical tasks by their effect on task-based efficiency metrics	100 RARP cases	Model obtained a Jaccard index of 0.85. Median difference between human rater and ML algorithm < 200 s for most steps
[37]	Luongo et al.	Automating the recognition and categorization of suturing gestures for needle driving attempts	2395 live suturing videos for identification and 511 live suturing videos for classification	Models identified a gesture with AUC 0.88 and classified the gesture with AUC 0.87
[38]	Kiyasseh et al.	Using a unified deep learning framework to recognize surgical phase, classify surgical gestures, and assess surgical skills	86 surgical videos of the NS step of RARP by 15 surgeons, 60 videos of RARP from 8 surgeons, 27 videos of RAPN from 16 surgeons, 78 videos of VUA from 19 surgeons	Models achieved AUCs between 0.857 and 0.898 for phase recognition, between 0.680 and 0.899 for gesture classification, and between 0.797 and 0.880 for surgical skills assessment in multi-center data validation
[39•]	Ma et al.	Automated prediction of 1-year erectile function with surgical gesture sequences	80 nerve-sparing RARP across 2 international medical centers	Models performed better than traditional clinical features in predicting 1-year erectile function (team 1: AUC 0.77, 95% CI 0.73–0.81; team 2: AUC 0.68, 95% CI 0.66–0.70)

*AUC* area under the curve, *DL* deep learning, *MAE* mean absolute error, *ML* machine learning, *NS* nerve spare, *PLND* pelvic lymph node dissection, *RAPN* robot-assisted radical nephrectomy, *RARP* robot-assisted radical prostatectomy, *VUA* vesicourethral anastomosis

### Brachytherapy/Radiation

AI also has been studied in the context of nonsurgical treatment planning for prostate cancer. In a study done by McIntosh et al., a ML model was trained to make plans for external radiation therapy (RT) [40]. When evaluated by a third blinded clinician, 89% of the ML-generated RT plans were deemed clinically acceptable, and 72% were chosen in head-to-head comparisons over human-generated RT plans. The median amount of time needed for the full RT planning procedure decreased by 60.1% (118 to 47 h). Interestingly, though the ML-generated plan performed well in simulation, the treating physician’s choice of the consensus-reviewed, quantitatively superior ML RT plans at the deployment phase decreased by 21%. These results highlight the fact that even in the presence of expert blinded review, retrospective or simulated evaluation of ML approaches may not be an accurate representation of algorithm acceptance in a real-world clinical situation when patient care is at risk.

Low-dose-rate prostate brachytherapy treatment takes place by implantation of small radioactive seeds in—and sometimes adjacent to the prostate gland—under the guidance of transrectal ultrasound images. In the planning process, it is standard practice to draw a line that closely resembles the genuine prostate boundary. To obtain a planned goal volume, the border is then dilated in relation to the clinical recommendations. This manual contouring is a laborious task with a significant amount of observer variability. To combat this, Nouranian et al. proposed an efficient learning-based multi-label segmentation algorithm to achieve clinically acceptable instantaneous segmentation results for seed implantation planning [41].

### Patient-Informed Treatment Decision-Making

AI has also been used in the informed decision-making process. Auffenberg et al. developed a web-based system that allows patients to input their own specific information to generate treatment options [42]. It was trained using random forest model processing data from 7543 prostate cancer patients covering a variety of different therapies (i.e., active surveillance, radical prostatectomy, radiation therapy, and androgen-deprivation therapy) for newly diagnosed prostate cancer patients. Both patients and doctors may benefit from using this tool to help them make informed decisions about the appropriate therapy that is best tailored for each individual patient.

## Prognostics

### Survival/Mortality Prediction

ML models are capable of rapidly processing large sums of clinical data which allows for increased prognostic capabilities in prostate cancer. Bibault et al. used an ML model that could predict the risk of prostate cancer mortality within 10 years of diagnosis and consisted of 30 clinical features with an accuracy of 0.98. Gleason score, PSA at diagnosis, and age had the largest impact on the model’s prediction [43].

With advances in precision oncology, there is an increasing trend toward personalizing the management of diseases to better fit the individual patient. Koo et al. created an online support tool using a long short-term memory ANN model to predict survival outcomes based on initial treatment modalities using data from 7267 patients which provided accurate, individualized survival outcomes at 5 and 10 years [44]. To better understand disparities among prostate cancer patients of different racial backgrounds, efforts are being made to investigate the importance of race and other nonbiological factors on prostate cancer-specific mortality. Hanson et al. used the SEER database and applied a random forest model to analyze different variables and interactions across 4 major categories of factors crucial to prostate cancer mortality, tumor characteristics, race, health care, and social factors [45]. Ultimately tumor characteristics at diagnosis were found to be the most important factor for PCa mortality. While race was also found to be a significant predictor of PCa mortality, health care and social factors had just as important implications on PCa mortality. Zhang et al. used a prognostic ML model to screen for DNA methylation of gene targets and identified FOXD1 as a therapeutic target for patients that have a poor prognosis. Due to the heterogeneity among patients, it is unlikely a singular model will encompass every aspect that contributes to mortality prediction [46]. Lee et al. developed a Survival Quilts model that predicts and compares predictions to other leading models to help improve its accuracy for personalized prognostics [47]. Similar calibration efforts may offer improvements in personalized prognostics.

### Recurrence Prediction

In addition to mortality prognosis, recurrence risk prediction is investigated following radical prostatectomies. The recurrence in PCa patients after a radical prostatectomy often couples with a higher mortality rate. ML algorithms can demonstrate accuracy at higher rates than the previously used predictive nomograms in predicting the recurrence of prostate cancer. Tan et al. trained 3 ML models that outperformed traditional nomograms in predicting biochemical recurrence at 1, 3, and 5 years with the best model reaching an AUC of 0.894 for 5-year recurrence. This provides an alternative to tailored care in multimodal therapy [48]. As seen in the ML involved in mortality prognosis, population-based variations suggest more aggressive tumors in African American prostate cancer patients. Bhargava et al. trained a random forest model (AAstro ML) with an African American-specific stromal signature that outperformed clinical standard Kattan and CAPRA-S nomograms with AUCs of 0.87 and 0.77 [49]. Biochemical factors have a considerable effect in causing recurrence in PCa patients. Machine learning has also shown that it can accurately predict the biochemical recurrence of PCa after robot-assisted prostatectomy using magnetic resonance imaging (MRI) quantitative features. This has significant implications in optimizing treatment such as neoadjuvant or adjuvant therapies for patients.

## Conclusions

In conclusion, there is widespread potential of the implementation of AI in the field of urology, particularly in the diagnosis and treatment of prostate cancer. Many studies have shown that AI-powered systems can accurately detect prostate cancer and help predict patient outcomes, leading to higher potential for improved patient care. However, there are several limitations to the use of AI in medicine that must be considered. For example, AI systems rely on the quality and quantity of data that they are trained on and may not perform as well when applied to real-world situations that differ from the data that was used to initially develop and train these algorithms. In addition, there are concerns about the ethical implications of using AI in medical decision-making, as well as the potential for bias in the algorithms that drive these systems. While there are still limitations and challenges to the widespread adoption of AI in medicine, the available evidence suggests that AI has the potential to revolutionize the field of urology and improve patient outcomes in relation to prostate cancer. Further research is needed to fully understand the potential and limitations of AI in this field, and to develop strategies for implementing AI in a way that maximizes its benefits while minimizing potential risks. Ultimately, AI remains as a potential tool to be used by urologists or other specialists to help guide their clinical decision-making and has not yet reached a point that it can or should supplant trained clinical professionals.
